# Physics-Informed Optimization for the Sub-Feature-Scale Fabrication of Hollow Microneedles via Digital Light Processing

**DOI:** 10.3390/mi17060678

**Published:** 2026-05-29

**Authors:** Junhong Huang, Zhangzhe Xu, Shuo Wu, He Zhang, Guanzheng Liu, Bin Liu

**Affiliations:** 1 Guangdong Provincial Key Laboratory of Sensor Technology and Biomedical Instrument, School of Biomedical Engineering, Shenzhen Campus of Sun Yat-sen University, Shenzhen 518107, China; 2 Department of Otolaryngology, The Third Affiliated Hospital of Sun Yat-sen University, Guangzhou 510630, China; 3 State Key Laboratory of Robotics and Systems, Harbin Institute of Technology, Harbin 150001, China

**Keywords:** hollow microneedles, digital light processing, additive manufacturing, drug delivery

## Abstract

To overcome low bioavailability and high trauma in inner ear therapies, targeted delivery across the round window membrane (RWM) via hollow microneedles (HMNs) offers a promising solution. However, the fabrication of high-aspect-ratio, small-size HMNs remains challenging. This study demonstrates the successful fabrication of small-outer-diameter HMNs using a 10 μm resolution digital light processing (DLP) system. Finite element analysis (FEA) identified a double tangent-arc transition as the optimal structural design for minimizing stress concentration. To manage the heightened parameter sensitivity at sub-feature-scale fabrication, a corrected curing index (CCI) model was established via a physics-informed regression approach incorporating polymerization kinetics and nonlinear spatial intensity distribution, achieving high fitting accuracy (R^2^ > 0.96). Under optimized parameters, the fabricated HMNs possessed mean dimensions of 805.13 μm in height, 37.54 μm in inner diameter, and 79.36 μm in outer diameter. Compressive tests exhibited a robust structural strength of up to 141 mN per needle following post-curing. Combined in silico and in vitro experiments demonstrated excellent penetration performance. Furthermore, the HMNs achieved stable, pressure-dependent delivery with volumetric flow rates rising from 0.14 mL∙min^−1^ to 0.39 mL∙min^−1^ as driving pressure escalated from 50 kPa to 300 kPa, validating their functional capacity for controlled drug administration.

## 1. Introduction

Inner ear diseases, causing deafness, tinnitus, and vertigo, represent a leading cause of hearing impairment [1]. However, the inner ear is encased within the dense temporal bone, making surgical drug delivery highly challenging [2]. The current treatment methods could be divided into invasive therapy and non-invasive therapy. Invasive surgeries such as temporal bone drilling pose significant risks such as large trauma and prolonged recovery [3]. Non-invasive therapy such as systemic or tympanic administration exhibits low drug utilization, resulting in poor treatment outcomes [4]. Among various treatment approaches, round window membrane (RWM) injection is recognized as an effective and promising inner ear drug delivery method [5,6,7]. Nevertheless, the RWM’s occlusion and extremely small size (approximately 2 mm in diameter) [8] means that conventional syringe injection is difficult to localize and prone to causing significant trauma to the patient.

**Figure 1:**
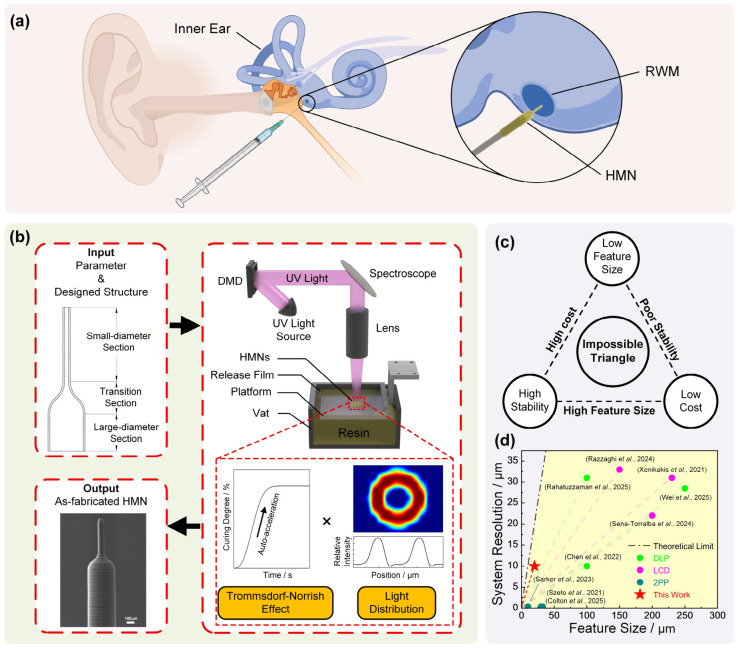
Schematic diagram of sub-feature-scale fabrication of polymeric hollow microneedles (HMNs) via digital light processing (DLP) printing. (**a**) Scenario of HMN penetrating the round window membrane (RWM). (**b**) Schematic diagram of the HMN fabrication process. (**c**) Schematic diagram of factors affecting HMN processing. (**d**) Comparison between this work and other works fabricate HMNs using photopolymerization methods [15,16,17,18,19,20,21,22,23].

Microneedle-based drug delivery represents a novel methodology that facilitates targeted and highly efficient therapeutic administration [9]. Compared to conventional delivery routes, microneedles bypass critical physiological hurdles such as hepatic first-pass metabolism and gastrointestinal degradation [10]. By penetrating physical barriers, microneedles establish microchannels for either drug delivery [11] or diagnostic sampling [12]. Among various microneedles, hollow microneedles (HMNs) emerge as a particularly promising alternative. HMNs feature internal channels that enable the continuous and precise delivery of macromolecular therapeutics [13], which have been applied in clinical scenarios such as ophthalmology [14] and otolaryngology [15] (Figure 1a). For instance, Szeto et al. [15] fabricated and utilized HMNs with an outer diameter (OD) of 100 μm and an inner diameter (ID) of 35 μm to perform RWM penetration in guinea pig models, which could significantly enhance drug permeability across the RWM to the inner ear. Furthermore, the created tissue damage during the penetration process is negligible due to the diminutive dimensions. Therefore, HMNs are a promising alternative for inner ear drug delivery due to the unique structural and functional characteristics.

Currently, small-scale HMNs are primarily fabricated from metallic, silicon-based, and polymeric materials. Metallic HMNs involve complex fabrication processes, making it difficult to achieve hollow isodiametric structures with high aspect ratio [24]. Small-scale silicon-based HMNs are typically fabricated using etching techniques [25]. However, they exhibit poor mechanical properties and are prone to brittle failure [26]. In contrast, polymeric materials offer superior biocompatibility, a reduced risk of fracture and more established fabrication pathways [27], thereby providing a viable foundation for the clinical translation of HMNs.

Existing fabrication methods for polymeric HMNs primarily include micromolding [28], laser machining [29], and 3D printing [30,31]. While micromolding and laser machining struggle to efficiently produce small-scale, isodiametric HMNs with high aspect ratio [28,29], polymer 3D printing technology offers design freedom and manufacturing flexibility. Consequently, this approach has garnered significant attention from researchers as a premier solution for advanced HMN fabrication [16,32,33]. Kumar et al. [34] utilized stereolithography (SLA) to fabricate HMNs with an OD of 439 μm and an ID of 189 μm, subsequently exploring their applications in transdermal gene delivery. Sena-Torralba et al. [21] employed a liquid crystal display (LCD) printing system to produce HMN arrays with an OD of 600 μm and an ID of 200 μm. Kouassi et al. [35] utilized fused deposition modeling (FDM) to fabricate HMN with an OD of 2000 μm and an ID of 1000 μm, corresponding to a relatively high diameter within HMNs. Due to the inherent resolution constraints of the printing principle, successfully fabricating sub-hundred-micron HMNs with high aspect ratio with traditional 3D printing methods like LCD, SLA or FDM techniques is still challenging.

To address these limitations, high-precision polymer 3D printing modalities like two-photon polymerization (2PP) and digital light processing (DLP), provide viable solutions for the fabrication of high-resolution HMNs. 2PP utilizes a femtosecond laser to induce localized polymerization within a focal volume, enabling the fabrication of complex structures with sub-micron sizes [36]. While 2PP can achieve high-precision fabrication of sub-hundred-micron HMNs, its high processing costs, low fabrication throughput and specific requirements for systems and materials limit widespread adoption.

DLP is a vat photopolymerization method that employs a digital micromirror device (DMD) to project entire layers of UV light onto a liquid resin, allowing for the rapid fabrication of high-resolution structures of approximately 1 μm [37] (Figure 1b). Compared with 2PP methods, DLP ensures adequate fabrication precision at a relatively low cost, making it the ideal candidate for balancing performance and cost [38]. Chen et al. [16] utilized a DLP printing system with 10 μm resolution to fabricate an array of HMNs with a base diameter of 600 μm and an aspect ratio of 2. Moussi et al. [32] demonstrated DLP-printed (2 μm resolution) reservoir-integrated HMN arrays composed of needles 400 μm high with OD of 120 μm (aspect ratio ≈ 3.3). These studies concluded that DLP is a viable candidate for the fabrication of small-scale HMNs. However, the aforementioned studies indicate that despite the potential of DLP, significant challenges remain in the fabrication of high aspect ratio, small-outer-diameter HMNs with precision close to the feature size, here termed as “sub-feature-scale precision”. In such a fabrication scenario, the critical local geometric elements and continuous intra-pixel curing gradients directly approach or constrain the system’s 10 μm × 10 μm physical pixel limits [39]. Fundamental investigation into fabrication mechanisms and systematic optimization of fabrication parameters remain scarce.

The fabrication of HMNs at the sub-hundred-micron scale faces dual challenges (Figure 1c). First, the resolution of DLP systems is more than an order of magnitude inferior to that of 2PP systems. Second, the requirement for an unobstructed internal hollow channel within a high aspect ratio structure further compounds the fabrication difficulty. To address these challenges, current research typically employs fabrication systems in which precision far exceeds the target feature size, thereby ensuring fabrication success rates and reducing iterative optimization costs (Figure 1d). However, this approach significantly increases fabrication costs and imposes higher demands on equipment performance. This study aims to enable the efficient and cost-effective DLP fabrication of small-outer-diameter, isodiametric HMNs with high aspect ratio through the optimization of processing parameters and analysis of photopolymerization theory. Furthermore, this work seeks to investigate low-cost DLP systems with sub-feature-scale precision when fabricating HMNs. Finally, the mechanical property and sample delivery performance of the fabricated HMNs are evaluated, offering insights into the development of polymeric HMNs and their application in biological tissue penetration and drug delivery.

## 2. Materials and Methods

### 2.1. Design of HMNs

The HMN structure was designed with three segments, including a small-diameter segment, a transitional segment and a large-diameter segment connected to a 30-gauge blunt needle to optimize mechanical and functional performance for biosampling and targeted drug delivery in inner ear therapy. Furthermore, three transitional geometries and three types of needle tip designs were evaluated.

The small-diameter segment was designed with a height of 800 μm or 400 μm with an OD of 80 μm, while the large-diameter segment was designed with an OD of 400 μm. A uniform wall thickness of 20 μm was maintained throughout the HMN structure. HMN arrays consisting of only the small-diameter and transitional segments were fabricated with an inter-needle spacing of 500 μm in a 5 × 5 configuration.

Finite element analysis (FEA) was conducted to optimize the transition geometries of HMN. All models used in FEA were constructed in SolidWorks 2019 (Dassault Systems, Paris, France). The mechanical simulation was employed using the Transient Structural module of ANSYS 2023 Mechanical (ANSYS Inc., Pittsburgh, PA, USA). Fixed support was added at the bottom of each HMN, and a load of 100 mN [40] was applied at the tip of HMN. The material properties of HMN were defined based on the manufacturer’s data of printing resin to ensure consistency. All simulation parameters and settings are comprehensively presented in Table S1.

### 2.2. Fabrication of HMNs

All HMNs were fabricated using a DLP 3D printing system (S140, Boston Micro Fabrication Material Technology Inc., Shenzhen, China) with the rigid photocurable resin (HTL resin, Boston Micro Fabrication Material Technology Inc., Shenzhen, China). The resin mainly consists of methacrylate oligomers and monomers as the precursor and diphenyl(2,4,6-trimethylbenzoyl)phosphine oxide (TPO) as the photoinitiator. The characteristics of the DLP system are presented in Table S2. Existing studies indicate that reducing layer thickness enhances axial mechanical strength; therefore, the minimum layer thickness of 10 μm was adopted [41]. The fabrication parameters of exposure time and exposure intensity were systematically investigated. Crucially, all other fabrication variables were held constant, including the platform movement speed and acceleration (0.5 mm/s and 1 mm/s^2^), detachment distance (4 mm) and leveling time (60 s/layer).

After printing, the fabricated HMNs were retrieved and cleaned by immersion in isopropanol (Guangzhou Chemical Reagent, Guangzhou, China) combined with 40 kHz ultrasonic treatment (F-031ST, Fuyang, Shenzhen, China) for 5 min. The HMNs were then vacuum-dried at room temperature. Post-curing was performed using a UV curing system (LS-1601, EFL, Suzhou, China) for 16 min.

### 2.3. Performance Characterization

To evaluate the functional performance of the fabricated HMNs, comprehensive characterization was conducted on the HMN with a 400 μm small-diameter segment height. Characterization included morphological analysis, mechanical testing, penetration assessment and in vitro drug delivery evaluation.

#### 2.3.1. Morphology

The surface morphology and tip structure of the HMN were examined with a scanning electron microscope (SEM) (JSM-IT800, JEOL Ltd., Tokyo, Japan). Prior to SEM observation, all samples underwent magnetron sputtering deposition of silver coating for 8 min using a magnetron sputtering coater (VTC300, SYMicrotec Co., Ltd., Shenyang, China). The dimensional characterization of HMNs were measured using an optical microscope (VHX-7000, Keyence, Osaka, Japan).

#### 2.3.2. Mechanical Properties

1. Destructive test

The mechanical performance of HMNs was tested using a universal testing machine (ZQ-990B, ZhiQu Precision Instrument Co., Ltd., Dongguan, China) to determine their maximum load-bearing capacity. The HMNs were fixed on the bottom plate. The top plate descends at a test speed of 1 mm/min to compress the HMNs [42]. Real-time force and displacement were obtained through the computer. The peak value was extracted from the force—displacement curve as the ultimate failure force of the HMN.

2. Penetration properties

In silico study of RWM penetration by the HMN was simulated using Abaqus (Dassault Systèmes, Vélizy-Villacoublay, France) with the Explicit Dynamics module. The analytical model comprised three components: a needle holder for applying force and directional constraints to the HMN, the HMN, and the RWM model. The RWM model employed an elliptical cylindrical geometry (70 μm thickness, 2.04 mm major axis and 1.81 mm minor axis). The RWM’s material properties included the density (1200 kg/m^3^) and hyperelasticity described by a second-order Ogden model [43]. All parameters of RWM were derived from the research of Liang et al. [8]. Mass scaling was judiciously applied to mitigate numerical oscillations during simulation. The mechanical response curve was generated by extracting reaction forces at the HMN base support.

Penetration tests of HMNs were performed at a speed of 1 mm/min using the universal testing machine. A single layer of Parafilm^®^ (PM996, Amcor plc, Zurich, Switzerland) with a thickness of approximately 150 μm was used as a tissue simulant for experimental validation [28].

#### 2.3.3. In Vitro Drug Delivery Study

Phosphate-buffered saline (PBS) was used to evaluate the drug delivery performance of the HMNs. The fabricated HMNs were affixed to 30-gauge blunt needles using a low-viscosity UV-curable adhesive (LOCTITE 3311, Henkel, Aachen, Germany), ensuring mechanical stability and fluidic connectivity. The assembled system was connected to a compressed air source, and the pressure was regulated. The delivered fluid was collected and measured in real time using an electronic balance (FA224C, readability: 0.1 mg, Lichen, Shaoxing, China). The corresponding volume was calculated based on fluid density, and a volume–time relationship was established.

## 3. Results and Discussion

### 3.1. Structure Optimization

**Figure 2:**
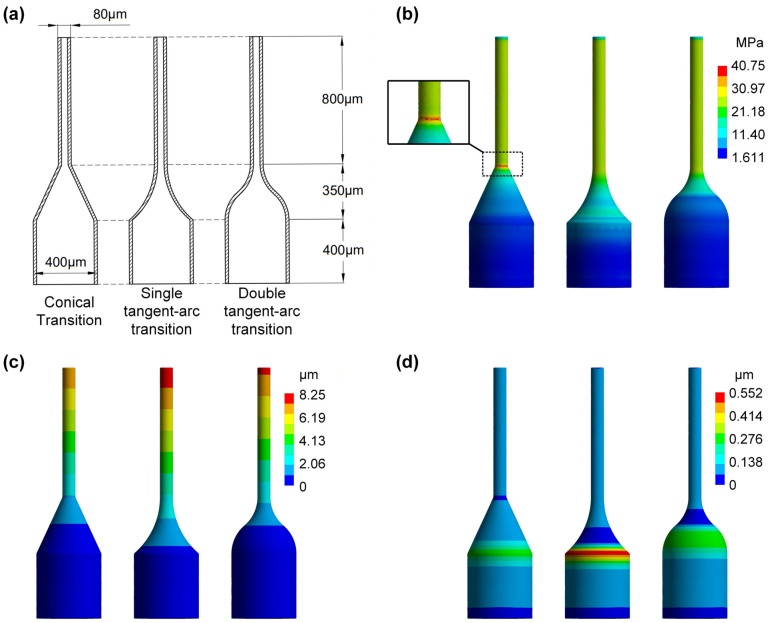
Mechanical simulation of polymeric HMNs with three types of transition segment. (**a**) Schematic diagrams of three types of HMNs. (**b**) Stress distribution contour plots of different HMNs. (**c**) Longitudinal deformation contour plots of the different HMNs. (**d**) Transverse deformation contour plots of the different HMN structures.

Three types of transition geometries were chosen to optimize the structure (Figure 2a). As illustrated in the stress distribution contours (Figure 2b), the conical transition exhibited a pronounced stress concentration at the junction segment, reaching a peak value of approximately 40.75 MPa. Such localized stress concentration significantly affects the mechanical stability of 3D-printed HMNs. Unlike monolithic fabrication, additive manufacturing processes inherently produce weaker interlayer bonding [44,45], making the conical transition design highly susceptible to fracture initiation at these locally stress concentration zones. In contrast, the arc-based transitions could effectively mitigate stress concentration through smooth curvature profiles.

Longitudinal deformation analysis showed that the region of maximum cumulative displacement is found to be more extensive in single tangent-arc design, indicating a relative reduction in transitional stiffness (Figure 2c). Transverse deformation mapping indicated that the longitudinal deformation of single tangent-arc design originated from insufficient structural support at the transition segment and reached a localized peak transverse deformation of 0.55 μm (Figure 2d), causing load transfer to the large-diameter section. In clinical scenarios, such transverse deformation may amplify needle bending under minor perturbations, potentially leading to premature bending failure or incomplete tissue penetration.

The results indicate that double tangent-arc transition eliminates observable stress concentration while providing higher transitional stiffness which significantly reduces overall deformation. Thus, the double tangent-arc transition effectively achieves a mechanical trade-off between reliability and rigidity for penetration applications.

### 3.2. Optimization of Processing Parameters and the Construction of CCI Model

The optimized HMNs with double tangent-arc transition segment were fabricated via DLP printing. The effect of exposure time and light intensity on structure dimension and performance fitting was quantitatively investigated.

**Figure 3:**
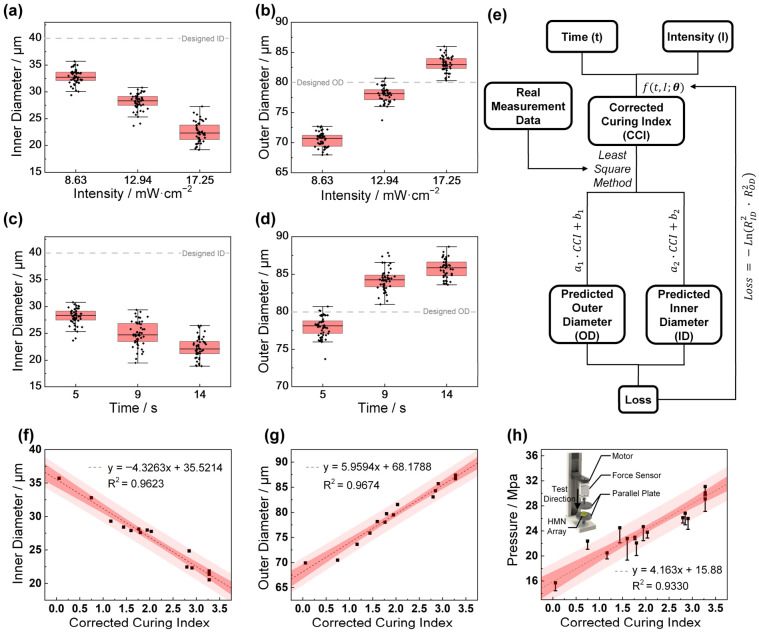
Investigation into HMN fabrication parameters. (**a**) IDs of HMNs under different light intensities. (**b**) ODs of HMNs under different light intensities. (**c**) IDs of HMNs under different exposure times. (**d**) ODs of HMNs under different exposure times. (**e**) Schematic illustration of the CCI fitting process using a physics-informed regression approach. (**f**) IDs of HMNs under different CCIs. (**g**) ODs of HMNs under different CCIs. (**h**) Failure pressure of the HMNs under different CCIs, with a corresponding schematic of the compression test.

The fabricated dimensions of the HMNs were presented via standard boxplots (whiskers representing the 1.5 × IQR range), overlaid with the complete set of individual measurement data points to explicitly show the full data distribution (Figure 3a–d). The HMN dimensions exhibit a consistent dependence on both light intensity and exposure time. Specifically, maintaining a constant exposure time (5 s) while increasing the light intensity from 8.63 to 17.25 mW∙cm^−2^ (Figure 3a,b) resulted in an ID reduction from 32.80 μm to 22.45 μm, accompanied by an expansion of the OD from 70.48 μm to 83.06 μm. A similar trend was observed in Figure 3c,d, where the ID decreased from 28.13 μm to 22.32 μm and the OD increased from 78.02 μm to 85.70 μm as the exposure time was extended from 5 s to 14 s at a fixed light intensity (12.94 mW∙cm^−2^).

The results consistently demonstrated positive correlations between both exposure parameters and dimensional deviations. Extending the exposure time or increasing the light intensity resulted in a higher exposure energy input [46,47]. This ultimately led to higher polymerization and greater discrepancies from designed dimensions, with a tendency for the ID to decrease and the OD to increase. These results indicate that in the context of sub-feature-scale DLP printing, the final HMN structures exhibit extreme sensitivity to processing parameters.

In photolithography and photopolymerization systems, exposure dose (Equation (1)) conventionally serves as a unified parameter combining exposure time and light intensity for process control, with its effects on material curing well established [48].

(1) Exposure Dose=t · I

where *t* is exposure time, *I* is light intensity.

Under typical UV photopolymerization conditions, identical exposure doses are expected to yield consistent fabricating outcomes [49,50]. However, experimental data from sub-feature-scale fabrication (Figure S1) revealed different dimensional outcomes under identical exposure doses [51]. This observation suggests that parameter interactions in sub-feature-scale fabrication require considerations beyond conventional exposure dose response relationships.

The fundamental principle of photopolymerization-based 3D printing lies in the polymerization of oligomers or monomers, where both light intensity and exposure time influence the final structural outcomes by modulating the curing process. According to the Trommsdorff—Norrish effect [52], the characteristic auto-acceleration of polymerization reaction rates over time plays a critical role in determining resin polymerization kinetics. As the Trommsdorff—Norrish effect exhibits consistent behavior for each material system, its impact on processing outcomes should be theoretically predictable [53]. To quantitatively describe this effect, the Avrami equation with undetermined empirical material coefficients was adopted as a phenomenological approach [54,55,56]:

(2) $$\alpha \left(t\right)=\;1-\exp\left[-a\cdot t^{b}\right]$$

where *α* is the curing degree of the material, *t* is exposure time, and *a*, *b* are empirical material coefficients.

Building upon this conceptual framework, Jiang et al. [56] further established that all coefficients in the Avrami equation (*a* and *b* in Equation (2)) maintain linear relationships with light intensity during photoinitiated polymerization. Under these conditions, the coefficients in modified Avrami equation takes the following form:

(3) $$a\left(I\right)=\;\theta_{1}\cdot I+\theta_{2}$$

(4) $$b\left(I\right)=\theta_{3}\cdot I+\theta_{4}$$

where *I* is light intensity and *θ*_1_, *θ*_2_, *θ*_3_, *θ*_4_ are the undetermined empirical constants related to material properties.

Meanwhile, the periodic microstructure of DMD arrays introduces diffraction effects in DLP systems [57,58], leading to image distortion. While spatial filtering can mitigate these distortions [59], inherent limitations of optical components persist, such as high-frequency spatial cutoff and stray light. These factors result in non-uniform intensity distribution across the projection field.

Based on the above two factors affecting sub-feature-scale DLP, a modified curing kinetics model was developed that incorporates nonlinear effects during DLP printing, termed the corrected curing index (CCI) formulation:

(5) $$CCI=f\left(t,I;\theta \right)=\left(1\;-\;e^{-\left(\theta_{1}I+\theta_{2}\right)\cdot t^{{(\theta }_{3}I+\theta_{4})}}\right)\;\cdot \;I^{\theta_{5}}$$

where *t* is exposure time, *I* is light intensity, and *θ*_1_, *θ*_2_, *θ*_3_, *θ*_4_, *θ*_5_ are the undetermined constants.

The experimental validation included systematic characterization of specimens fabricated across multiple combinations of exposure time and intensity parameters. In a typical 3D printing task utilizing a DLP system with specified system configuration and material properties, the curing degree of the material is intrinsically linked to the received exposure. To investigate this relationship, HMN arrays fabricated under varying printing parameters were characterized while omitting any post UV curing. The relevant experimental data are presented in Table S3.

Given the substantial number of parameters in the CCI formulation, a gradient descent-based regression approach with the loss function as the optimization criterion was employed (Figure 3e). The regression framework for sub-feature-scale DLP printing optimization is structured as an iterative, physics-informed regression loop that bridges processing parameters with geometric outcomes. The process begins by inputting exposure time and light intensity into the corrected curing kinetics function which derives a latent variable termed the CCI to represent the extent of polymerization. Utilizing the least square method, the CCI is linearly mapped to fit the OD and ID, respectively. During each iteration, the coefficient of determination (R^2^) is calculated by comparing the predicted and experimental values of ID and OD, as defined in Equation (6):

(6) $$R^{2}=1-\;\frac{\sum_{i=1}^{n}{\left(y_{i}-{\hat{y}}_{i}\right)}^{2}}{\sum_{i=1}^{n}{\left(y_{i}-\bar{y}\right)}^{2}}.$$

where n is total number of data points, $y_{i}$ is experimental value of the sample, ${\hat{y}}_{i}$ is fitted value of the sample and $\bar{y}$ is the mean value of all experimental observations.

The fitting performances are then calibrated against real measurement data to compute a composite loss function, defined by the summation of the coefficients of determination. The form of R^2^-based loss function is:

(7) $$Loss=\;-Ln(R_{ID}^{2}\cdot R_{OD}^{2}).$$

The logarithmic transformation of the R^2^ amplifies the absolute gradient as R^2^ approaches 1. Such a treatment imposes stronger feedback constraints on sub-optimal metrics. Consequently, this approach accelerates the convergence speed of the regression model (Figure S2). This loss is fed back into the CCI model to iteratively refine the empirical parameters. Initial regression using only curing kinetics yielded sub-optimal fits R^2^ value of approximately 0.908 for ID and 0.891 for OD. This discrepancy highlights the need to account for DMD-induced optical distortions and non-uniform intensity distributions. Also, comparative analysis revealed that incorporating nonlinear terms consistently improved model performance (Table S4).

By employing the CCI model to unify light intensity and exposure time, the R^2^ for the linear fit between the CCI and the fabricated HMN’s ID and OD reached 0.9623 and 0.9674, respectively (Figure 3f,g). Through the physics-informed regression approach, the specific mathematical expression of the CCI model was:

(8) $$CCI=f\left(t,I\right)=\left(1\;-e^{-\left(0.002I-0.0067\right)\cdot \;t^{\left(0.0503I+1.7633\right)}\;}\right)\cdot \;I^{0.4168}.$$

In summary, the mathematical modeling suggests that exposure time and light intensity influence the fabrication process through two primary mechanisms: (1) the combined Trommsdorff-Norrish effect of exposure time and intensity on photoinitiated polymerization kinetics, which governs curing process in the monomers/oligomers of photosensitive resin, and (2) optical path components’ modulation of DMD-reflected light intensity distribution at the curing plane.

It is worth noting that the dimensional discrepancies arising from the nonlinear curing kinetics and DMD-induced optical modulations are theoretically omnipresent across any cross-sectional geometry with physical boundaries, independent of the structural scale. In macro-scale fabrication, the error introduced by these spatial optical distortions is minuscule relative to the total part volume, rendering its impact negligible within standard engineering tolerances. However, as the structural feature sizes scale down, the ratio of this deviation to the nominal structural dimensions escalates, thereby emerging as a dominant factor. Furthermore, based on the CCI model, this phenomenon represents an intrinsic physical characteristic of the DLP, rather than a geometry-dependent anomaly exclusive to HMNs.

To characterize the intrinsic material properties of the HMNs independent of their specific geometries, compression tests were performed on the HMN arrays. The failure pressure measurements (Figure 3h) demonstrated the correlation with a linear fit to CCI, achieving an R^2^ value of 0.9330. This correlation may be attributed to the CCI model’s precise description of the curing degree, as the significant influence of the degree of polymerization on the mechanical strength of DLP-printed materials has been substantiated [60]. These results confirm that the CCI model not only reflects dimensional deviations of HMNs processed near the resolution limit of the DLP system but also explains the influence of curing degree on mechanical properties in this context. The CCI framework effectively accounts for performance variations in the fabricated components under these processing conditions, establishing its capability to interpret product performance deviations.

### 3.3. Performance Characterization

The optimized fabrication parameters, 5 s exposure time and 12.94 mW∙cm^−2^ light intensity, determined by the regression approach model, were adopted to fabricate the HMNs which produce an OD of about 80 μm and implemented design compensation for the ID dimensions (from 40 μm to 50 μm). Subsequently, a 16 min post-processing UV curing process was performed.

#### 3.3.1. Morphology

**Figure 4:**
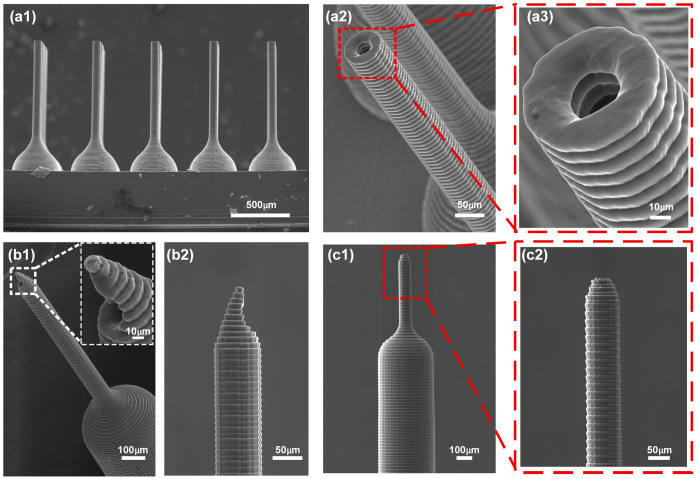
Morphology characterization of HMNs. (**a1**) Side-view SEM image of the HMN array. (**a2**) SEM image of the flat-tip HMN. (**a3**) The magnified view of the flat-tip HMN. (**b1**) Side-view SEM image of the side-hole tip HMN. (**b2**) The magnified view of the side-hole tip HMN. (**c1**) Side-view SEM image of the conical tip HMN. (**c2**) The magnified view of the conical tip HMN.

Figure 4 presents the SEM images of HMNs featuring various tip structures. As shown in Figure 4(a1), the HMN array exhibits a highly ordered configuration that remains consistent with the initial design specifications. The measured height of the HMN is 805.13 μm, demonstrating precise axial control. Figure 4(a2,a3) provide magnified views of an individual HMN, where the OD and ID are approximately 79.36 μm and 37.54 μm, respectively. Furthermore, the SEM images reveal robust interlayer bonding within the HMNs, characterized by distinct laminar features inherent to the 3D printing process and a high degree of interlayer structural consistency. However, due to fabrication system constraints, the HMNs exhibit a higher sidewall roughness compared to the RWM-penetrating HMNs fabricated using 2PP [15], representing a clear morphological limitation in precision surface control. The typical hollow structure of the HMN is shown in Figure S3.

To address specific application requirements, the HMNs enable targeted design adjustments, such as reducing the needle height for enhanced mechanical stability or integrating sharpened tips to improve penetration performance. Two additional needle tip geometries consisting of the side-hole (Figure 4(b1,b2)) and conical (Figure 4(c1,c2)) tips were designed and fabricated to complement the HMN structure. The side-hole tip HMNs featured a tip diameter of approximately 29.5 μm, whereas the conical tip HMNs measured approximately 47.3 μm. Both tip geometries have been confirmed to effectively reduce penetration force [61,62]. Morphology characterization confirmed that the fabricated structures matched the design specifications. Furthermore, the structural integrity of the sharpened tips was successfully verified.

#### 3.3.2. Mechanical Performance

A typical clinical application of microneedles is penetration. Therefore, reducing penetration force and minimizing penetration-related tissue damage are key considerations in clinical application. Both theoretical and experimental methods were applied to analyze the penetration and mechanical behavior of the HMNs.

**Figure 5:**
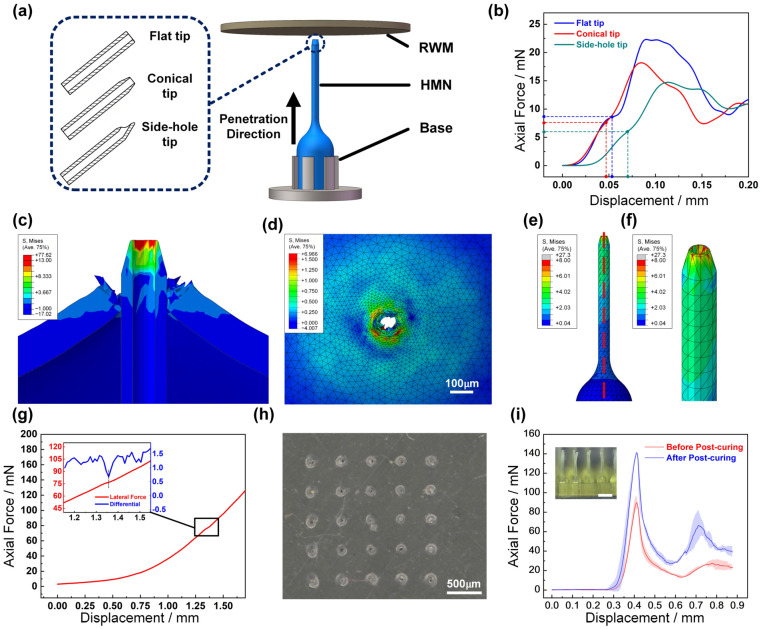
Mechanical properties of HMNs. (**a**) FEA analytical model of the HMN penetrating the RWM. (**b**) Penetration force—displacement curves of HMNs with different tips. (**c**) Stress distribution contour plot after complete penetration by the HMN. (**d**) Induced damage and stress distribution contour plot in the RWM following HMN penetration. (**e**) Stress distribution contour plot of the HMN. (**f**) Enlarged view of the needle tip stress distribution. (**g**) Penetration performance of the fabricated HMN with illustration shows the force inflection point. (**h**) Micrograph of the Parafilm^®^ damage after HMN penetration. (**i**) Destructive performance of the fabricated HMNs before and after post-curing and micrograph of HMN array after compression (scale bar: 500 μm).

FEA was employed to compare the penetration behaviors of these distinct tip geometries when penetrating the human RWM. The components of analytical model were shown in Figure 5a. The perforation event was identified by a distinct inflection point in the force—displacement curve, after which the HMN was retracted. Conical and side-hole tip designs significantly reduced penetration forces compared to non-tipped (flat-tip) counterparts. As shown in Figure 5b, conical tip HMNs require greater penetration force (7.92 mN) but cause minimal membrane deformation (48 μm), whereas side-hole tip HMNs require less penetration force (6.18 mN) but result in greater membrane displacement (71 μm). The side-hole tip exhibits a lower penetration force primarily due to its significantly smaller equivalent tip radius, which generates higher localized stress concentration at lower loads. However, the side-hole tip requires a larger displacement to achieve penetration because its asymmetrical geometry lacks the direct axial stress concentration of the conical tip, leading to a prolonged elastic stretching phase where the RWM undergoes more extensive deformation before reaching its failure strain. This difference indicates that tip designs should be optimized based on specific application requirements. Simulation results for conical tip HMN penetration indicate that the HMN is capable of penetrating the RWM of the inner ear (Figure 5c). The diameter of the penetration cavity corresponds to approximately 1.41 times the OD of the HMN (Figure 5d). The HMN tips remained intact following penetration (Figure 5e,f), exhibiting only localized stress concentration at the apex with no structural buckling along the needle shaft.

Penetration test confirms an average penetration force of approximately 76.8 mN per needle for the HMN array (Figure 5g). Beyond the fact that the Parafilm^®^ thickness is more than double that of the RWM, the additional resistance arising from the multi-point contact of the HMN array further accounts for the higher measured penetration force compared to the FEA of single HMN. This demonstrates that the HMNs are capable of performing effectively in more demanding penetration scenarios than those typically encountered with the RWM. Following the penetration test, regularly patterned micro-perforations were observed on the Parafilm^®^ layer, exhibiting a spatial distribution that precisely correlates with the HMN array configuration (Figure 5h). This outcome demonstrates the robust mechanical integrity of the fabricated HMNs, as every individual needle within the array fully penetrated the membrane without structural failure.

Flat-tip HMNs were applied to evaluate the destructive properties influenced by the post-curing process. As shown in Figure 5i, the results indicate an average critical 89.8 mN buckling force of individual HMNs for the HMN array before post-curing. Buckling indicates the structural failure of the HMNs. Post-curing induces a 1.57-fold increase in buckling force to 141 mN on average by completing the cross-linking initiated during printing. These results are mainly attributed to the incomplete polymerization of the resin during the fabrication process. Consequently, the degree of cross-linking was further enhanced through a post-curing step, which significantly enhanced the mechanical performance of the HMNs [63]. Post-compression microscopy revealed HMN curvature, confirming plastic deformation as the primary failure mode, with negligible structural changes observed in transitional segments. This irreversible deformation emphasizes the necessity of post-curing processes to enhance the mechanical performance to accommodate the requirements of RWM penetration.

#### 3.3.3. Drug Delivery Performance

**Figure 6:**
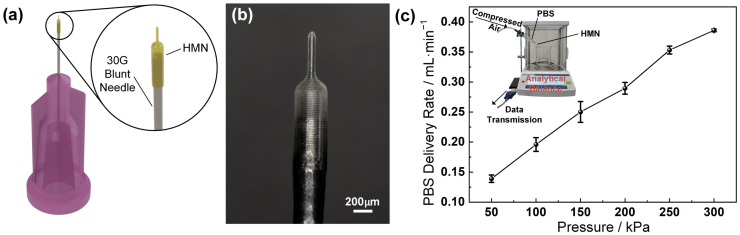
Drug delivery performance characterization of HMN. (**a**) Schematic diagram of the connection between the 3D-printed HMN and a 30-gauge blunt needle. (**b**) Micrograph of the connection between the HMN and the blunt needle. (**c**) Characterization of the pressure-driven PBS delivery using individual HMN.

Drug delivery performance evaluation experiments were conducted on individual HMNs (Figure 6a). The micrographs reveal a robust and tight integration between the HMN and the blunt needle, characterized by a seamless and smooth transition at the interface (Figure 6b).

To evaluate the drug delivery performance and controllability of the fabricated HMNs, the delivery rate of PBS was investigated under varying pressures. The quantitative relationship between the applied driving pressure and the resulting PBS delivery rate is presented in Figure 6c. A near-linear positive correlation was observed across the entire evaluated pressure spectrum from 50 kPa to 300 kPa. Specifically, at a relatively low initiating pressure of 50 kPa, the delivery system yielded a steady flow rate of approximately 0.14 mL∙min^−1^. As the driving pressure was increased to 150 kPa and 300 kPa, the PBS delivery rate monotonically increased to 0.25 mL∙min^−1^ and 0.39 mL∙min^−1^, respectively. The relatively large internal diameter enables continuous and stable drug administration. These results validate the functional efficacy of the fabricated HMNs for precise drug delivery applications.

## 4. Conclusions

This study demonstrates the successful sub-feature-scale fabrication of HMNs via a DLP printing system. FEA identified the double tangent-arc transition as the optimal design for maximizing mechanical strength and deformation resistance. To manage the heightened sensitivity of sub-feature-scale fabrication to processing parameters, a CCI model was established through a physics-informed regression approach, incorporating the Trommsdorff—Norrish effect into HMN dimensions and mechanical performance. Morphological characterization of the HMNs showed that the optimized parameters could fabricate small-outer-diameter, high-aspect-ratio HMNs with a height of 805.13 μm, ID of 37.54 μm, and OD of 79.36 μm. Mutual validation through in silico FEA simulations and in vitro penetration experiments demonstrates the robust mechanical performance of the HMNs for RWM applications. Mechanical performance test exhibited a 1.57-fold increase in compressive strength up to 141 mN following post-curing. Furthermore, the fabricated HMNs demonstrated a stable drug delivery performance with the volumetric flow rate increasing from 0.14 mL∙min^−1^ to 0.39 mL∙min^−1^ as the driving pressure escalated from 50 kPa to 300 kPa, validating their functional capacity for controlled drug administration in inner ear therapy.

Regarding the biocompatibility of the fabricated HMN, although the current commercial resin is not certified as fully biocompatible according to the manufacturer’s safety data sheet, advanced surface modification techniques such as gold plating [64] or spray-coating of functional polymers [65] have been widely documented to enhance the surface safety profile effectively. Although this study demonstrates a controllable fabrication method to produce high-aspect-ratio polymeric HMNs for RWM penetration, their biocompatibility, biosafety, fluid delivery performance and structural stability under more realistic in vivo biological environments still demand further investigation in our future work.

## Data Availability

The original contributions presented in this study are included in the article/Supplementary Materials. Further inquiries can be directed to the corresponding author.

## Supplementary Materials

The following supporting information can be downloaded at: https://www.mdpi.com/article/10.3390/mi17060678/s1, Table S1: Material properties and FEA simulation settings in this research; Table S2: System characteristics of DLP system; Table S3: Experimental fabrication parameters and corresponding dimensional measurements of the fabricated HMNs; Table S4: Fitting performance of different function combinations; Figure S1: Comparison of HMN wall thickness fabricated under identical exposure doses. Box plots represent sample size *n* = 50 per group. Statistical significance between the designated adjacent processing groups was evaluated via paired-sample *t*-tests, indicated as *** *p* < 0.001; Figure S2: Comparison of convergence speeds of different loss functions; Figure S3: Top-view SEM image of an individual HMN.

## Abbreviations

The following abbreviations are used in this manuscript:

HMN	hollow microneedle
RWM	round window membrane
DLP	digital light processing
CCI	corrected curing index
OD	outer diameter
ID	inner diameter
SLA	stereolithography
LCD	liquid crystal display
2PP	two-photon polymerization
UV	ultraviolet
FDM	fused deposition modeling
FEA	finite element analysis
TPO	diphenyl(2,4,6-trimethylbenzoyl)phosphine oxide
SEM	scanning electron microscope
DMD	digital micromirror device
PBS	phosphate-buffered saline
R^2^	coefficient of determination
