# Supplementary material for: Physics-Informed Optimization for the Sub-Feature-Scale Fabrication of Hollow Microneedles via Digital Light Processing
Source: Micromachines (Basel). 2026 May 29;17(6):678. doi: 10.3390/mi17060678 (PMC13303251; doi:10.3390/mi17060678)
Supplement: Supplementary file 1 [file micromachines-17-00678-s001.zip › micromachines-4312472-supplementary.pdf]

Article

Physics-Informed Optimization for the Sub-Feature-Scale Fabrication of Hollow Microneedles via Digital Light Processing

Junhong Huang, Zhangzhe Xu, Shuo Wu, He Zhang, Guanzheng Liu and Bin Liu

Table S1. Material properties and FEA simulation settings in this research.

FEA model	Parameter / Condition	Value
General	HMN density	1.17 g/cm^3
	HMN Young's modulus	2397 MPa
	HMN tensile strength	71.5 MPa
	Poisson's ratio	0.375
Mechanical performance	Fixed support	Bottom of HMN
	Load	HMN tip, 100 mN
Penetration simulation	RWM size	Short axis: 1.81 mm<br>Long axis: 2.04 mm<br>Thickness: 70 μm
	RWM density	1.2 g/cm^3
	RWM hyperelasticity	Second-order Ogden Model<br>μ1: 0.43 MPa<br>α1: 1.02<br>μ2: 0.42 MPa<br>α2: 1.04
	Mesh unit type	C3D10M
	Penetration speed	3 mm/min
	Contact setting	Surface-to-surface contact
	Contact pair faces (Master-slave Surfaces)	Base - HMN bottom<br>HMN tip - RWM<br>HMN body - RWM
	Failure criteria	Maximum logarithmic strain > 0.20

Table S2. System characteristics of DLP system.

Parameter	Value
Pixel size	10 μm×10 μm
UV wavelength	405 nm
Maximum optical power	110 mW·cm^{-2}

Table S3. Experimental fabrication parameters and corresponding dimensional measurements of the fabricated HMNs.

Exposure time / s	Light intensity / mW·cm^{-2}	CCI	Average ID /μm	Average OD /μm
1	12.94	0.0552	35.70	69.90
3	21.59	2.0327	27.77	81.54
5	8.63	0.7465	32.80	70.48
3.35	17.25	1.5994	27.89	78.16
3.75	17.25	1.9460	27.98	79.49
5	12.94	1.7648	28.13	78.02
5	17.25	2.7971	22.45	83.06
7.5	8.63	1.4403	28.41	75.87
9	8.63	1.7985	27.61	79.72
9	12.94	2.8460	24.86	84.29
9	17.25	3.2771	21.86	86.69
10	6.47	1.1650	29.30	73.63
14	12.94	2.9071	22.32	85.70
14	17.25	3.2775	21.37	86.89
19	8.63	3.2775	20.54	87.40

Table S4. Fitting performance of different function combinations.

$R^2({}^{ID}/_{OD})$	Power function	Exponential function	Logarithmic function	/
Avrami function	${}^{0.9623}/_{0.9674}$	${}^{0.9302}/_{0.9373}$	${}^{0.9161}/_{0.9419}$	${}^{0.9079}/_{0.8907}$
/	${}^{0.2288}/_{0.3611}$	${}^{0.2156}/_{0.3432}$	${}^{0.2174}/_{0.3618}$	/

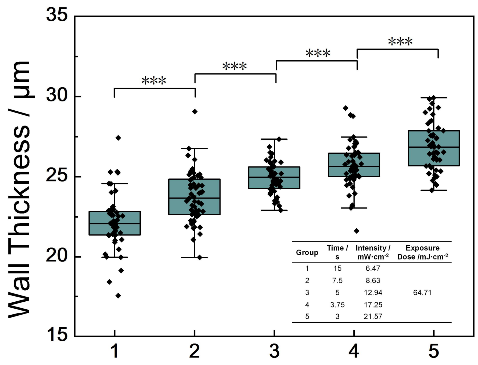

Figure S1. Comparison of HMN wall thickness fabricated under identical exposure doses. Box plots represent sample size n = 50 per group. Statistical significance between the designated adjacent processing groups was evaluated via paired-sample t-tests, indicated as ***$p < 0.001$.

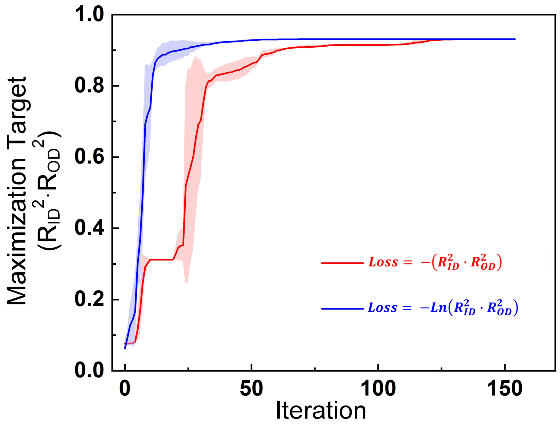

Figure S2. Comparison of convergence speeds of different loss functions.

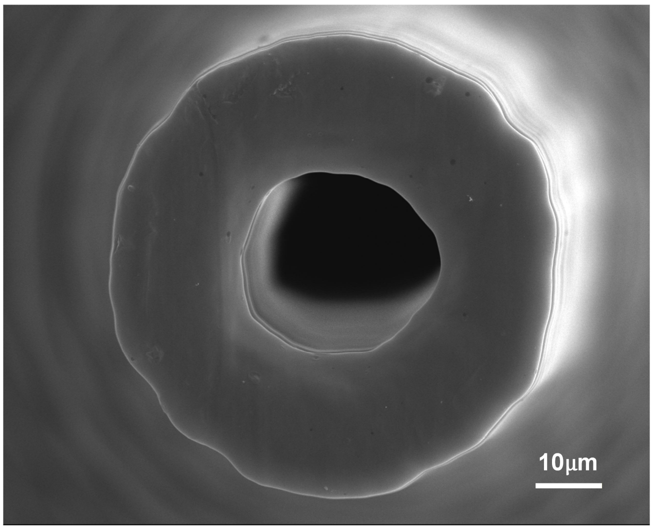

Figure S3. Top-view SEM image of an individual HMN.
